# Printed vs. Digital Patient Information Leaflets—Improving Patient Information Delivery for Enhanced Engagement in Trauma and Orthopaedic Surgery: A Quality Improvement Project

**DOI:** 10.7759/cureus.93991

**Published:** 2025-10-06

**Authors:** Jefferson George, Indhu Poomalai, Clare Wildin

**Affiliations:** 1 Trauma and Orthopaedic Surgery, University Hospitals of Leicester NHS Trust, Leicester, GBR

**Keywords:** carbon footprint, cost-effective practice, digital health technology, fracture clinic, qr code, trauma and orthopaedic surgery

## Abstract

Introduction

The Green Surgery Report, endorsed by the Royal College of Surgeons of England, states that improving the quality of patient care goes hand in hand with sustainability. Patients arriving at the fracture clinic are regularly provided with printed patient information leaflets (PILs) as part of their ongoing care. Current digital technology enables the use of sustainable forms of patient information, such as quick response (QR) codes and digital records. This quality improvement project explored the sustainability potential of improving patient accessibility to information through the use of digital technology.

Methods

Two prospective Plan-Do-Study-Act cycles were carried out from July to October 2024. All patients arriving at the fracture clinic of the Leicester Royal Infirmary were included. Novel questionnaires were used to assess patient experience in receiving PILs in both digital and print forms. Change was introduced by creating QR codes for relevant PILs. Patient preference, cost analysis, and impact on carbon footprint were evaluated.

Results

In the first cycle with 50 patients, only 31 (62%) reported reading the paper or printed information leaflets, and 38 (76%) preferred receiving information in a digital form or via a QR code. All patients aged ≥80 years preferred printed information, while patients in the age range of 15-65 years predominantly preferred digital information. Following the implementation of QR codes in the second cycle, which included 20 patients, 16 (80%) found it easier to access and save digital PILs for future reference. A cost-effectiveness analysis revealed an estimated annual cost saving of £1600 and a reduction in CO_2_ emissions of ~252 kg.

Conclusion

This project demonstrated measurable benefits towards healthcare information delivery and greener surgery through using QR codes for PILs while simultaneously improving patient engagement.

## Introduction

Patient information leaflets (PILs) are routinely used in the fracture clinic to convey critical information to patients about their condition, treatment, and rehabilitation [1]. Approximately one-third of ED attendances with musculoskeletal injuries are followed up by the orthopaedic fracture clinic service [2] and are provided with PILs as part of their ongoing care. PILs are also used routinely in surgical decision-making [3] to help patients consent for surgery and provide information on post-operative instructions and recovery. PILs serve as vital decision aids that enhance patient engagement and promote shared decision-making [4].

There is a growing trend towards reducing reliance on printed and paper documents in the National Health Service (NHS) through the digitalisation of healthcare delivery. The underlying aim is to improve access to up-to-date healthcare information while promoting sustainability at all times [5]. The Green Surgery Report (UKHACC, 2023) advocates that improving the quality of care goes hand in hand with sustainability [6]. In line with this, the NHS has also committed to becoming the world’s first national health service to reach net zero carbon emissions by 2040 for all directly controlled emissions (NHS Carbon Footprint) and by 2045 for all emissions the NHS can influence (NHS Carbon Footprint Plus) [7]. Specific initiatives such as the NHS Business Services Authority’s 2022/2023 target to reduce office paper use by 45% underscore the urgency of moving away from traditional practices [8]. In this context, printed PILs are an easily achievable area of reform, aligning with broader sustainability goals. 

The use of printed paper in healthcare settings contributes directly or indirectly to environmental degradation through resource consumption, waste generation, and the release of greenhouse emissions. Each A4 sheet of printer office paper contributes to about 5 g of CO_2_ per sheet [9]. With each PIL being at least 2 to 3 sheets of A4 paper, the use of PILs in printed form contradicts the NHS's commitment to sustainable practices. 

In this quality improvement project, we evaluated the feasibility of replacing printed PILs with digital PILs accessed via quick response (QR) codes. Previous work in other specialties has shown that QR codes can be a viable alternative to traditional PILs through ease of access, storage, and reduced environmental burden [10,11]. This project aimed to assess current practice, implement QR codes to commonly used PILs, and evaluate the usability and acceptability of this intervention in the fracture clinic setting of trauma and orthopaedic surgery. In addition, we also estimated the potential for cost savings and reduction in carbon emissions and explored the barriers to adoption of practice. 

Objectives

Primary Objective

The study aimed to evaluate patient preference and engagement with QR code-based vs. paper-based PILs in a fracture clinic setting.

Secondary Objectives

The study aimed to assess the cost and carbon footprint implications of replacing paper PILs with QR codes and evaluate patient confidence and barriers in using QR codes.

## Materials and methods

This quality improvement project was conducted at the Fracture Clinic and Trauma Triage at Leicester Royal Infirmary, University Hospitals of Leicester, between July 2024 and October 2024. The project was structured around two prospective audit cycles using the Plan-Do-Study-Act (PDSA) methodology. The first PDSA cycle (July-September 2024) focused on understanding patient preferences for digital vs. paper PILs and collecting baseline data on the use of printed leaflets. The second PDSA cycle was carried out from September to October 2024 after the implementation of QR codes to evaluate patient experience and the practicality of using QR codes compared to traditional printed leaflets. This project followed a structured framework in accordance with the Standards for QUality Improvement Reporting Excellence (SQUIRE 2.0) guidelines [12].

The data collected included patient demographics, such as age and gender, and details of the presenting injury. Structured questionnaire surveys assessed utilisation of printed PILs, preference for paper vs. QR code formats, confidence in scanning and saving QR codes, and perceived advantages or disadvantages of each format (Tables 1, 2). Carbon footprint and cost reduction for the NHS through the use of digital PILs were also estimated. All patients who were issued PILs were eligible for inclusion, regardless of age, gender, or injury type. Patients who declined participation or were unable to complete the questionnaire due to any reason such as cognitive impairment or language barrier were excluded. Convenience sampling was used, aligned to accommodate the schedules of the resident doctors leading the project. The sample size was determined based on the project's timeframe and staff availability. No formal sample size calculation was performed, as this was a quality improvement project. 

**Table 1:** First cycle survey questionnaire Table credits: Jefferson George (author) QR, Quick response

No.	Question	Answer
1.	Were you given a patient information leaflet at your initial visit?	Yes/No
2.	Did you find the information given in the leaflet useful?	Yes/No
3.	Did you read the printed leaflet given to you?	Yes/No
4.	Do you use a smartphone/does your carer have a smartphone?	Yes/No
5.	Would you prefer to receive information about your care in digital form (e.g., through a QR code)?	Yes/No
6.	How do you prefer the information be provided to you - if paper, why?	1. Easier to read 2. More tangible 3. No access to digital devices 4. Prefer to have a physical copy
7.	How do you prefer the information be provided to you - if digital (QR code), why?	1. Easier to access anytime 2. Environmentally friendly 3. Reduces clutter 4. Easier to store and retrieve
8.	How confident are you in using a digital leaflet (QR code)?	Likert scale 1 to 5

**Table 2:** Second cycle survey questionnaire Table credits: Jefferson George (author) QR, Quick response

No.	Question	Answer
1.	Are you familiar with QR codes?	Yes/No
2.	What did you use to scan the QR code?	1. Camera 2. QR scanner app 3. Did not work 4. Other
3.	How easy was it for you to scan the QR code and access the patient information leaflet?	Likert scale 1 to 5
4.	Did you require assistance in using the QR code?	Yes/No
5.	Were you able to save or bookmark the digital information for later reference?	Yes/No
6.	How would you compare using the QR code to receiving a paper leaflet?	1. I prefer using the QR code 2. I prefer receiving a paper leaflet 3. Both formats work equally well for me
7.	Any suggestions/feedback on QR codes for patient information leaflets ?	

**Figure 1:**
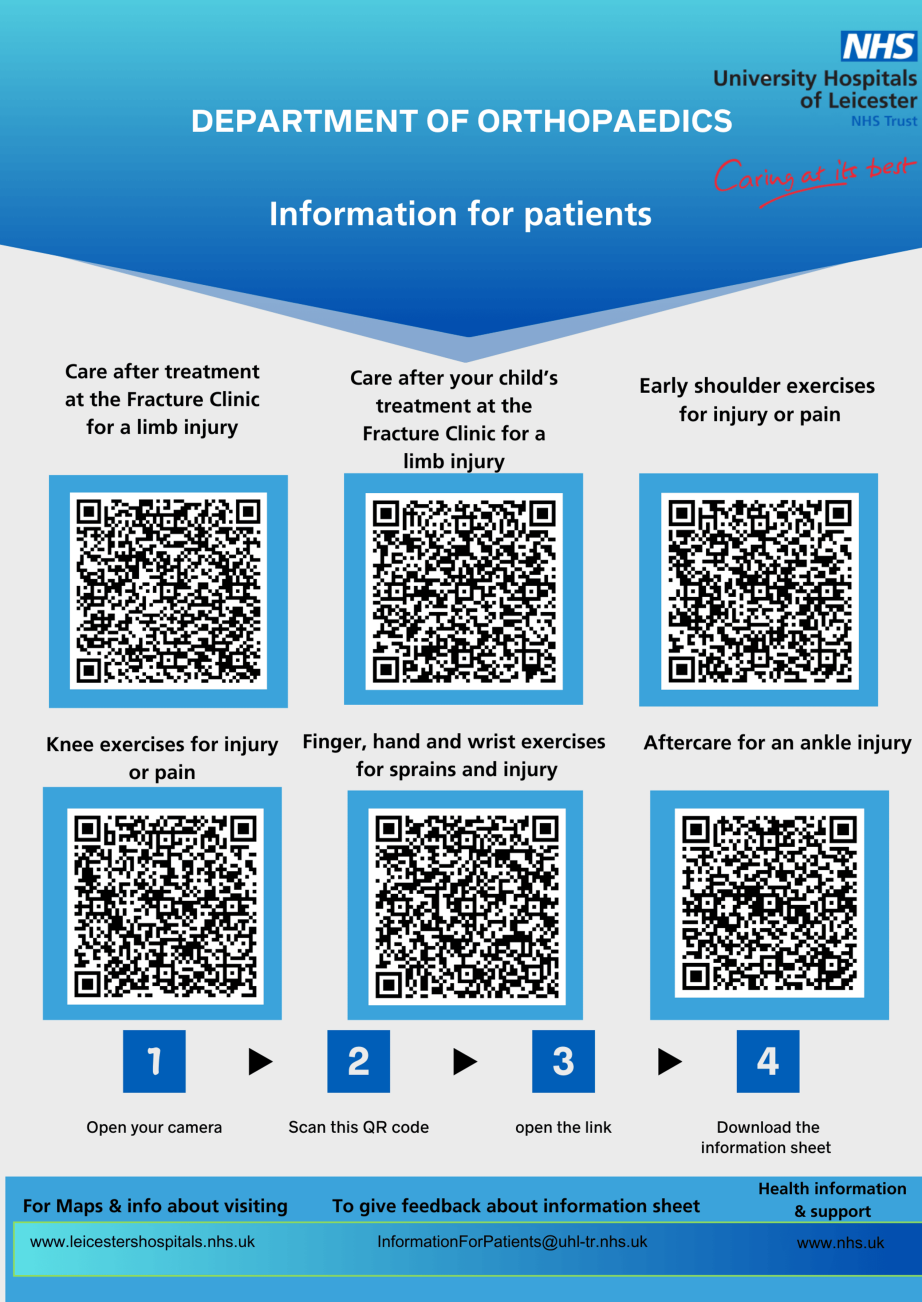
QR codes for six commonly used PILs QR codes for six commonly used PILs at University Hospitals of Leicester NHS Trust are available on the hospital’s public access domain [13]. Image credits: Indhu Poomalai (author) PIL, Patient information leaflet; QR, Quick response

The most commonly used PILs (sourced from the University Hospitals of Leicester “Your Health” website) were converted into QR codes (Figure 1). The intervention was communicated to consultants, registrars, resident doctors, and nurses through emails and meetings. A consolidated QR code poster (Figure 1) was displayed in consultation rooms and trauma triage areas and additional copies were provided to nursing staff for use during consultations.

Data from both cycles were collated and compared to assess patient preference and engagement with digital and printed PILs. Descriptive statistics were used to summarise findings, with subgroup comparisons by age group to identify trends in digital preference. Pearson's Chi-square (χ2) test was used to analyse categorical variables, while Fisher's exact test was used where the patient number was small. A p-value <0.05 was considered significant. The measure of association was determined using the contingency coefficient (C). The project was registered with the hospital audit department and followed all internal policies governing the execution of a quality improvement project.

Measurable outcomes for this project included the proportion of patients who preferred digital information over printed leaflets, stratified by age group; whether patients received a PIL; and the perceived usefulness of the leaflet. Additional outcomes included the proportion of patients who reported reading the leaflet completely, ownership of a smartphone, familiarity with QR codes, whether assistance was required to scan the QR code, and whether patients were able to bookmark or save the digital leaflet for later use. The estimated reduction in printing costs, based on the number of paper PILs avoided and the projected environmental benefit in terms of paper saved and carbon emissions reduced, was included in the final analysis. All outcome measures were derived from the survey questionnaire and patient demographics. An audit log was maintained in the hospital’s internal server for project documentation. Being a quality improvement project, the success of the project was arbitrarily defined as at least 50% of patients preferring the QR code PILs with no major barriers reported and estimated reductions in cost and carbon footprint of at least 30%. The project aimed to answer the question: Are QR code-based PILs a feasible alternative to paper PILs in a fracture clinic setting?

## Results

Patient demographics

Across both cycles, a total of 70 patients attending the fracture clinic were randomly surveyed and included in the project (Table 3).

**Table 3:** Demographics and distribution of injuries Data were represented as n (%). *The Chi-square test was used to compare gender distribution. **Fischer’s exact test was used to compare age groups and injury distribution.

	1st cycle (n=50)	2nd cycle (n=20)	Test statistic value	p-value
Gender				
Male	23 (46%)	12 (60%)	0.63	0.427*
Female	27 (53%)	8 (40%)		
Age groups				
<18 years	9 (18%)	4 (20%)	0.177	0.981**
18-65 years	25 (50%)	9 (45%)		
66-79 years	12 (24%)	5 (25%)		
≥80 years	4 (8%)	2 (10%)		
Injury				
Hand and wrist	21 (42%)	10 (50%)	0.408	0.999**
Upper limbs: Shoulder and elbow	8 (16%)	3 (15%)		
Hip	2 (4%)	0		
Knee	2 (4%)	0		
Foot and ankle	9 (18%)	4 (20%)		
Paediatric injuries	5 (10%)	2 (10%)		
Miscellaneous	3 (6%)	1 (5%)		

First PDSA cycle: July-September 2024

Of the 50 patients included in the first cycle, only 31 (62%) patients reported reading the paper PILs previously given. About 38 (76%) patients preferred QR code-based digital PILs, quoting the following reasons: 29 (75%) found it easier to access and retrieve, 10 (25%) said it reduces clutter, and 10 (25%) found it to be an environmentally friendlier option. Of the 38 (76%) patients who preferred QR codes, 37 (97%) rated their confidence in using QR codes as 4 or 5 on the Likert scale, and all expressed willingness to switch to digital formats. Of the 12 (24%) patients who preferred paper leaflets, the main reasons were being easier to read and the desire for a tangible copy. Around 45 (90%) of included patients owned a smartphone. All patients in the ≥80 years age group preferred printed leaflets, while patients aged 18-65 years predominantly preferred digital information.

A significant association between age group and preference for digital PILs (χ² = 22.16, p = 0.00006) was observed in the first cycle. Because of the small number of patients in the ≥80 years age group, Fisher's exact test was performed after collapsing categories into ≤65 years vs. >65 years, which confirmed the association (odds ratio = 0.04, p = 0.0000099). The strength of the association, measured by the contingency coefficient (C = 0.55), was moderate, indicating that younger patients were significantly more likely to prefer digital formats, whereas older patients strongly favored paper leaflets. There was no significant association between age groups and whether a leaflet was received (χ² = 2.43, p = 0.488), its perceived usefulness (χ² = 3.18, p = 0.786), or whether it was read completely (χ² = 4.68, p = 0.585). However, owning a smartphone was significantly associated with age group (χ² = 8.65, p = 0.034), with only 50% of patients ≥80 years reporting access to a smartphone compared with over 90% of younger patients (Table 4).

**Table 4:** Analysis of first cycle survey results by age group Data were represented as n (%). **Fischer’s exact test was used to analyse preference for digital information. *Chi-square test was used for all other analyses.

	Yes	No	Test statistic value	p-value
Preference for digital information				
Age 65 years	27 (54%)	7 (27%)	0.04	0.0000099** (significant)
Age >65 years	2 (4%)	14 (28%)		
Leaflet received				
Age <18 years	5 (10.0%)	3 (6.0%)	2.43	0.488*
Age 18-65 years	22 (44.0%)	4 (8.0%)		
Age 66-79 years	8 (16.0%)	4 (8.0%)		
Age ≥80 years	3 (6.0%)	1 (2.0%)		
Perceived usefulness of leaflet				
Age <18 years	7 (14.0%)	1 (2.0%)	3.18	0.786*
Age 18-65 years	20 (40.0%)	5 (10.0%)		
Age 66-79 years	7 (14.0%)	2 (4.0%)		
Age ≥80 years	3 (6.0%)	2 (4.0%)		
Leaflet read completely				
Age <18 years	3 (6.0%)	5 (10.0%)	4.68	0.585*
Age 18-65 years	19 (38.0%)	7 (14.0%)		
Age 66-79 years	7 (14.0%)	5 (10.0%)		
Age ≥80 years	2 (4.0%)	2 (4.0%)		
Own a smartphone				
Age <18 years	7 (14.0%)	1 (2.0%)	8.65	0.034* (significant)
Age 18-65 years	24 (48.0%)	2 (4.0%)		
Age 66-79 years	10 (20.0%)	2 (4.0%)		
Age ≥80 years	2 (4.0%)	2 (4.0%)		

Second PDSA cycle (September-October 2024)

In the second cycle, after introducing QR codes to access digital PILs, 16 (80%) patients were familiar with QR codes and were able to save the leaflet for future reference. In terms of usability, all these patients reported finding it easy or very easy to access digital PILs. The most common method used to scan QR codes was the use of phone cameras, with 14 (70%) patients, followed by 2 (10%) patients using QR code scanning apps. No assistance was required by patients familiar with QR codes to access digital PILs. When comparing QR code leaflets with printed versions, 13 (65%) patients expressed a preference for QR codes, while 4 (20%) preferred paper leaflets and 3 (15%) reported that both formats were equally acceptable.

As observed in the first cycle, familiarity with QR codes was significantly associated with age (χ² = 9.44, p = 0.0239). Younger patients ≤65 years found QR codes easier to scan (χ² = 14.84, p = 0.0215), did not require assistance (χ² = 9.44, p = 0.0239), and were consistently able to bookmark the information (χ² = 9.44, p = 0.0239) compared to older patients (Table 5).

**Table 5:** Analysis of second cycle survey results by age group Data were represented as n (%). *Chi-square test was used.

	Yes	No	Test statistic value	p-value
Familiar with QR codes				
Age 65 years	12 (60%)	1 (5.0%)	9.44	0.0239*
Age >65 years	4 (20.0%)	3 (15%)		
Assistance required to scan QR codes			9.44	0.0239*
Age 65 years	12 (60.0%)	1 (5.0%)		
Age >65 years	4 (20.0%)	3 (15.0%)		
Bookmark digital information				
Age 65 years	12 (60.0%)	1 (5.0%)	9.44	0.0239*
Age >65 years	3 (15.0%)	4 (20.0%)		

Carbon footprint and cost saving estimation

An average of 700 PILs are given out each month in the fracture clinic of Leicester Hospital, with each leaflet being 2-3 sheets of A4 regular white printed paper. This translates to approximately £176 per month or £2000 per year. An A4 laser-printed paper currently produces 6 g of CO_2_ equivalent emissions, which translates to approximately 30 g of CO_2_ per PIL [14]. About 16 (80%) patients in the second PDSA cycle used QR codes in place of paper leaflets. Extrapolating this figure over a 12-month period suggests a potential for reduction in approximately 8400 printed PILs annually, resulting in savings of over £1600 and a decrease of 252 kg of CO₂ emissions.

## Discussion

This quality improvement project demonstrated that QR code-based digital PILs are a feasible and acceptable alternative to printed leaflets in trauma and orthopaedic surgery. A high patient preference for digital information through QR codes was found. QR code-based PILs were found to be easy to use and accessible for the majority of patients. Similar results of increased accessibility and reduced reliance on print material have been reported in other specialties such as urology [11], ophthalmology [15], and rheumatology [16]. Upton et al. showed similar benefits in acute cancer care, where QR codes were effective tools in providing up-to-date information reliably [10]. The results of this project extend the evidence to orthopaedic settings by offering a sustainable alternative to printed PILs and support the wider adoption of digital transformation initiatives as recommended in the Green Surgery Report (UKHACC, 2023) [6].

The secondary goal of this project was to assess its impact on environmental sustainability. On average, five orthopaedic surgeons, consultants, and registrars run clinics daily, each seeing 20-30 patients, in addition to those attending trauma triage. Patients across all age groups with a range of fractures and injuries attend the service. As part of routine practice, patients are provided with printed PILs, averaging 700 leaflets distributed per month. A significant potential for reduction in paper use, translating to over £1600 in cost savings and a reduction of 252 kg in carbon emissions, was demonstrated at the conclusion of the project. To provide perspective, this is equivalent to CO_2_ emissions from 642 miles driven by an average gasoline-powered passenger vehicle or from the charging of 20,373 smartphones [17]. A similar sustainability potential was noted by Nikookam et al., evaluating the use of digital PILs in dermatology. By reducing emissions and expenditure, transitioning to QR codes contributes to the NHS’s long-term environmental target and financial efficiency [18]. 

An unexpected finding from the survey in the first PDSA cycle was that a significant proportion of patients reported not using the printed PILs provided. This highlighted the potential gap between resource distribution and patient engagement. Oktay et al. [19] observed similar trends in their study on healthcare information and concluded that many patients prefer on-demand access to healthcare information and ignore printed material unless immediately relevant. Printed PILs may not always be effective in patient education and engagement.

The preference for digital PILs was strongly associated with age. The main barrier identified was digital unfamiliarity, highlighting the need for optional printed formats to maintain inclusivity. All patients aged 80 years and above preferred paper PILs, while younger patients in the age group of 18-65 years favoured digital formats. Comparable conclusions were noted by Sharara and Radia [20], who found that enhanced engagement with digital methods was largely restricted to tech-literate younger patients. Although digital PILs through QR codes are a promising alternative, this finding highlights the need for a hybrid approach to information delivery to promote equitable access to information. Digital PILs can be leveraged for younger digitally confident patients, and printed PILs can be continued to be used for older populations.

The strength of this project was its real-world applicability, with measurable benefit in a busy clinical environment. However, the project has its limitations. The second cycle had a relatively smaller sample size. This reduces the generalisability of the findings. A formal sample size calculation was not carried out before project commencement, as this was a quality improvement project. There is scope for selection bias, as patients not familiar with QR codes may have declined to participate. Selection bias may also have been present due to the use of convenience sampling, time constraints, and variable clinic workflows. The impact on information retention and patient outcomes was beyond the remit of this project and was not evaluated. This was another methodological limitation due to the project’s design.

## Conclusions

Building on these findings, future initiatives may include a larger sample size, different clinical areas, and the exploration of parameters such as retention and patient outcomes. Embedding QR codes in discharge summaries and patient correspondence could enhance scalability. Further work should also focus on bridging the digital literacy gap observed in older patients.

This project emphasises the need to incorporate digital information delivery through tools such as QR codes into routine clinical workflows. QR codes can be a valuable adjunct to patient information delivery through their ease of access and environmental sustainability. While QR codes cannot fully replace printed PILs, they offer a scalable hybrid solution that aligns with NHS sustainability goals. 
